# Stage IV pleomorphic carcinoma of the lung without recurrence for 6 years: a case report

**DOI:** 10.1186/s40792-017-0310-6

**Published:** 2017-02-21

**Authors:** Naoko Miura, Ryo Mori, Tomoyoshi Takenaka, Koji Yamazaki, Seiya Momosaki, Sadanori Takeo

**Affiliations:** 1 Department of Thoracic Surgery and Clinical Research Institute, National Kyushu Medical Center Hospital, Fukuoka, Fukuoka Japan; 2 Department of Pathology and Clinical Research Institute, National Kyushu Medical Center Hospital, Fukuoka, Fukuoka Japan

**Keywords:** Pleomorphic carcinoma, Oligometastasis, Metastasectomy

## Abstract

**Background:**

Pleomorphic carcinoma is a rare primary lung carcinoma that occurs at a rate of about 0.3%. Even with complete resection, the tumor usually recurs aggressively, resulting in a poor prognosis. Herein, we report a case of advanced pleomorphic carcinoma of the lung who had a long survival time after resection of the primary and metastatic sites.

**Case presentation:**

A 48-year-old man was admitted to our hospital due to abdominal pain. Systemic examination revealed a lung mass on the right and a tumor in the jejunum. Surgical resection of both tumors revealed pleomorphic carcinoma of the lung with metastasis to the jejunum. Follow-up after 6 years showed that the patient remained recurrence-free, without the need for additional postoperative treatment.

**Conclusions:**

A vigorous treatment strategy that included surgery had the potential to offer long-term survival, despite an advanced pleomorphic carcinoma with distant metastasis to other organs. Reports on more similar cases are needed to evaluate the value of this treatment option.

## Background

The mainstream treatment strategy for advanced non-small cell lung cancer (NSCLC) is systemic chemotherapy. Surgery or radiotherapy is usually employed for cases with early stage and for the purpose of reducing local symptoms caused by the tumor. On the other hand, pleomorphic carcinoma is a rare tumor that consists approximately 0.3% of primary lung tumors. It is aggressive and recurrence is common even after complete resection in the early stage.

In this report, we described a case of a patient with advanced pleomorphic carcinoma of the lung and who survived for a long time after resection of the primary and metastatic sites.

## Case presentation

A 48-year-old man was admitted to our hospital due to pain and tenderness in the upper and central part of the abdomen when fasting. There were no pertinent symptoms. His past medical history included benign prostate hyperplasia. He was a current smoker for 42 pack-years. Physical examination revealed nothing significant except for tenderness in the upper and central part of the abdomen.

**Fig. 1:**
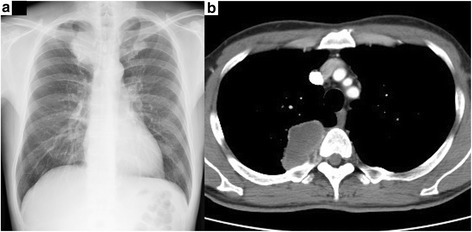
Radiologic imaging of the chest of a 48-year-old man with pleomorphic carcinoma of the lung. **a** Chest radiograph reveals a 6-cm shadow in the right upper lung field. **b** Chest computed tomography reveals a 6.5-cm tumor in the right upper lobe with invasion to the chest wall

**Fig. 2:**
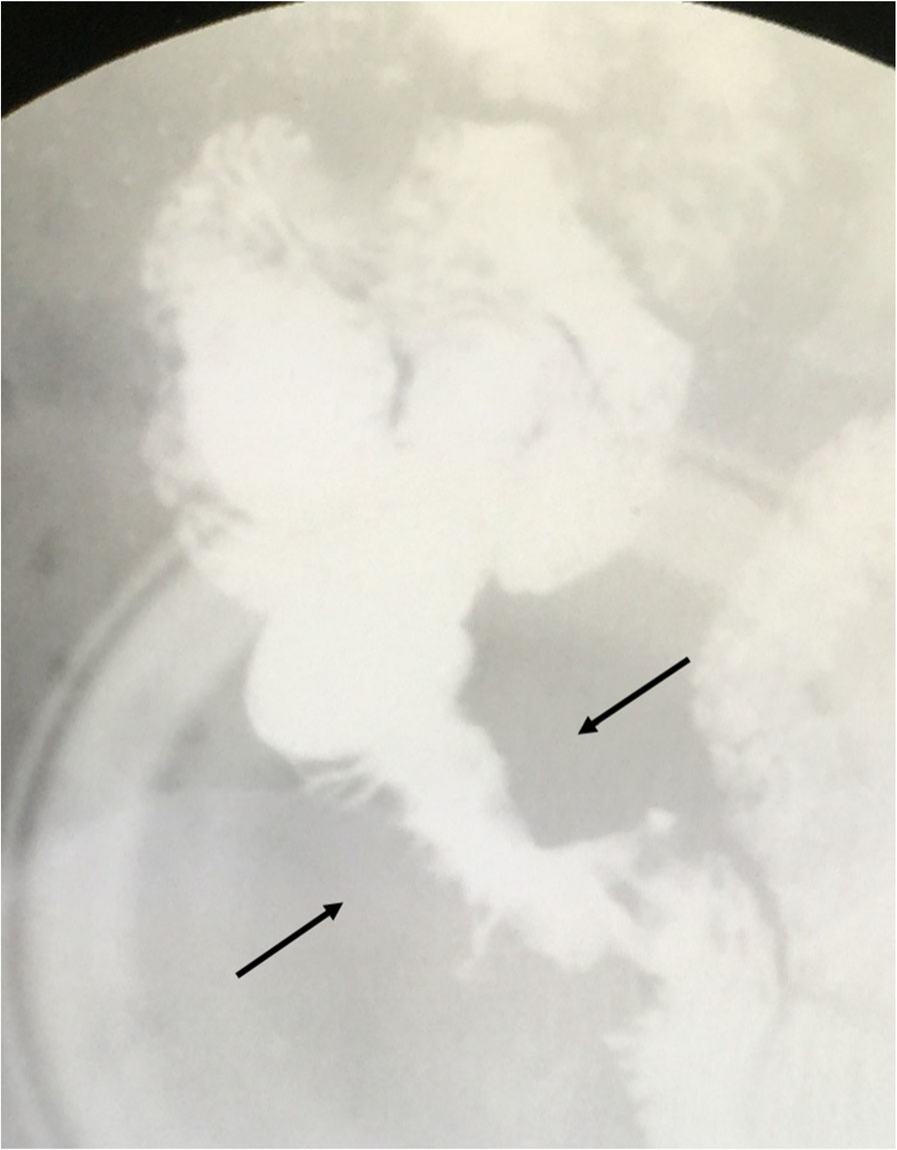
Intestinal fluoroscopy in a 48-year-old man with pleomorphic carcinoma of the lung. Stenosis in the distal part of the jejunum is shown (*arrows*)

Abdominal computed tomography (CT), upper gastrointestinal endoscopy, and complete colonoscopy revealed no abnormality. However, chest X-ray revealed a massive shadow in the right upper lung field (Fig. 1a), which was confirmed by chest CT showing a 65-mm mass in the right upper lobe with chest wall invasion (Fig. 1b). ^18F^-Fluorodeoxyglucose (FDG) positron emission tomography-CT showed abnormal accumulation of FDG in the lung tumor, as well as in a portion of the jejunum. Intestinal fluoroscopy revealed stenosis without obstruction in the distal part of the jejunum (Fig. 2). There was no evidence of lymph node involvement and distant metastases other than the jejunum. The clinical diagnosis was right lung cancer with metastasis to the jejunum. Transbronchial lung biopsy revealed no evidence of malignancy. The laboratory data revealed slight elevation in gamma GTP (80 IU/L) and C-reactive protein (0.78 mg/dL). The levels of tumor markers like carcinoembryonic antigen, cytokeratin-19 fragments, and pro-gastrin-releasing peptide were not elevated. Because the metastatic tumor in the jejunum was symptomatic and was localized in a single organ, surgery was planned for both the primary and metastatic sites. Partial resection of the jejunum was performed initially, followed by right upper lobectomy with chest wall resection within a month. The patient had an uncomplicated postoperative course.

**Fig. 3:**
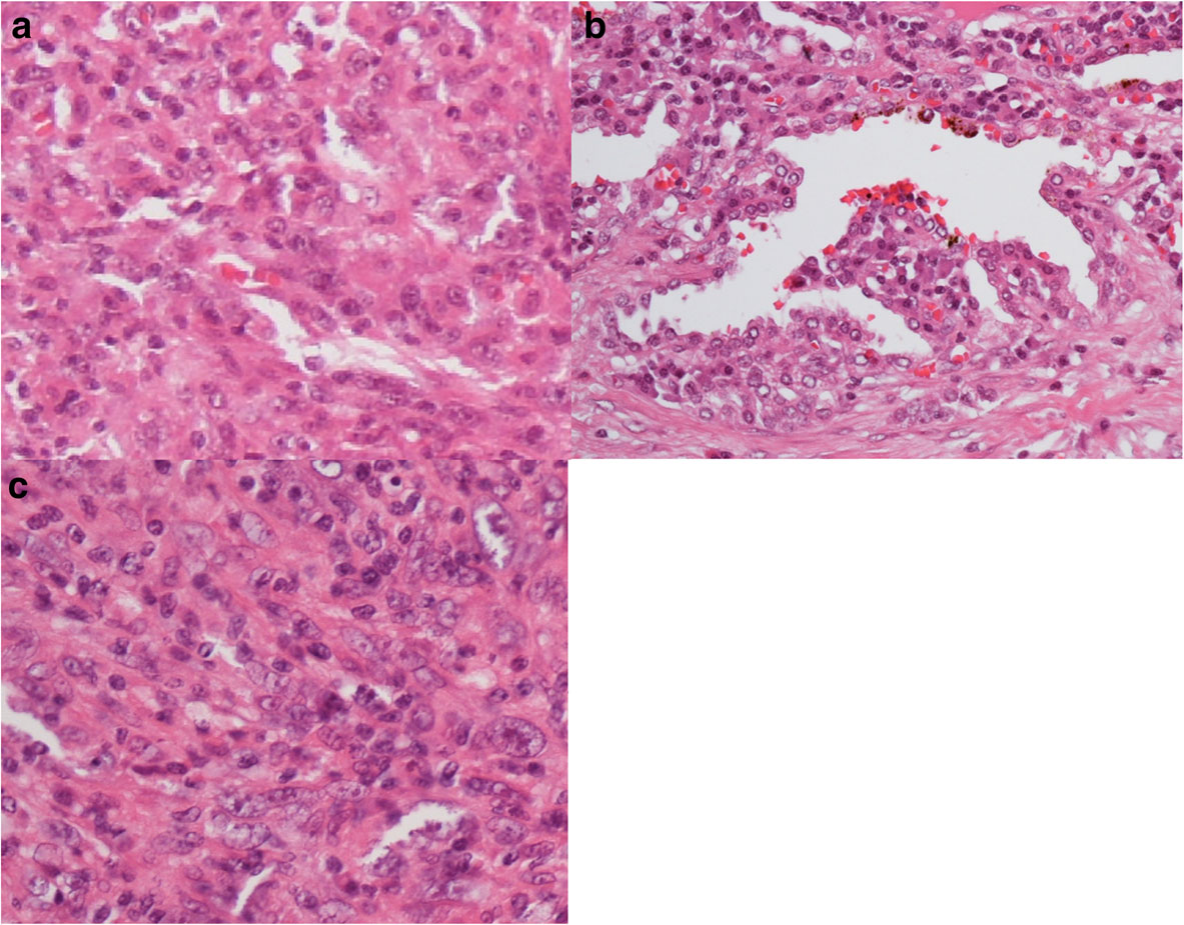
Pathologic findings in a 48-year-old man with pleomorphic carcinoma. Evaluation of specimens from both **a** the right upper lobe of the lung and **c** the jejunum shows atypical spindle cells with irregular and hyperchromatic nuclei with invasive proliferation and interlacing patterns of arrangement. **b** Epithelioid cells with tubular differentiation were also noted in the lung specimen (stained with hematoxylin and eosin, ×400)

Pathologic analysis of the lung tumor revealed atypical spindle cells with irregular and hyperchromatic nuclei invasively proliferating and arranged in interlacing patterns (Fig. 3a). Epithelioid cells with tubular differentiation (components of adenocarcinoma) were also noted in a small part of the lung specimen (Fig. 3b). Immunohistochemical studies revealed that the tumor cells were positive for vimentin, AE1/AE3, and TTF-1 but negative for c-Kit, alpha SMA, S-100, and CD34. The pathologic diagnosis was pleomorphic carcinoma of the lung (Fig. 3a, b). The tumor in the jejunum was similar in both morphologic and immunohistochemical analyses, confirming a metastasis from the pleomorphic carcinoma of the lung (Fig. 3c).

Since the patient did not want to receive adjuvant chemotherapy, we advised careful observation. The patient remained alive without recurrence for 6 years.

### Discussion

Pleomorphic carcinoma is one of the subtypes of sarcomatoid carcinoma among the four other subgroups, including spindle cell carcinoma, giant cell carcinoma, carcinosarcoma, and pulmonary blastoma. It is defined as a poorly differentiated NSCLC (squamous cell carcinoma, adenocarcinoma, or large cell carcinoma) containing spindle cells and/or giant cells, or a carcinoma that comprises spindle or giant cells alone, in at least 10% of the tumor [1]. Pleomorphic carcinoma occurs in only 0.1–0.4% of all lung carcinomas and generally has a more aggressive clinical course than the other types of NSCLC. According to the Japanese Joint Committee of Lung Cancer Registry data, its 5-year overall survival was 20–40%, whereas that of the other histologic types was 61.4% [1–4]. For such aggressive cancers, multimodality therapy needs to be considered, but the optimal treatment strategy remains undefined because of its rarity. The effective regimen for pleomorphic carcinoma, which is often diagnosed in the advanced stage, is not well established at the moment; in addition, adjuvant chemotherapy does not appear helpful.

Massive coagulation necrosis and lymph node metastases were identified as prognostic factors for a poorer outcome of pleomorphic carcinomas [2, 3]. However, pleomorphic carcinoma with pN0 has poorer prognosis than other variants of NSCLC with pN0 [3]. Frequent vascular invasion is the cause of this unfavorable prognosis, even in early-stage disease.

Some reports showed that a vigorous treatment strategy that included surgery had the potential to offer long-term survival, despite an advanced pleomorphic carcinoma with distant metastasis to other organs. In NSCLC, careful patient selection for complete resection of both primary and metastatic sites can result in a favorable outcome. Hasumi et al. reported on 35 surgically resected NSCLC with oligometastasis and concluded that patients without lymph node metastases had long-term survival [5]. For pleomorphic carcinoma, Aokage et al. reported two patients who survived for over 4 years after resection of the primary site and the gastric metastasis [6] and Yamanashi et al. reported a patient with brain metastasis who was successfully treated by brain metastasectomy followed by chemotherapy and resection of the primary lesion [7]. Similar to our present case, these reported pleomorphic carcinoma patients did not have regional lymph node metastases by both clinical and pathologic diagnoses. It is important to note that this unexpectedly good outcome should not become a rationale for surgical metastasectomy in all cases. However, complete primary lung tumor resection with metastasectomy might be a treatment option for pleomorphic carcinoma in node-negative patients.

## Conclusions

We reported a rare case of advanced pleomorphic lung carcinoma with metastasis to the jejunum who was successfully treated by surgery alone. Node-negative patients with oligometastasis might be candidates for surgery. Reports on more similar cases are needed to evaluate the value of this treatment option.

## Abbreviations

CT: Computed tomography
FDG: ^18F^-Fluorodeoxyglucose
NSCLC: Non-small cell lung cancer
